# Bicyclic Boronates as Potent Inhibitors of AmpC, the Class C β-Lactamase from *Escherichia coli*

**DOI:** 10.3390/biom10060899

**Published:** 2020-06-12

**Authors:** Pauline A. Lang, Anete Parkova, Thomas M. Leissing, Karina Calvopiña, Ricky Cain, Alen Krajnc, Tharindi D. Panduwawala, Jules Philippe, Colin W. G. Fishwick, Peteris Trapencieris, Malcolm G. P. Page, Christopher J. Schofield, Jürgen Brem

**Affiliations:** 1 Department of Chemistry, University of Oxford, Chemistry Research Laboratory, Oxford OX1 3TA, UK; pauline.lang@chem.ox.ac.uk (P.A.L.); t.leissing@googlemail.com (T.M.L.); karina.calvopinatapia@chem.ox.ac.uk (K.C.); alen.krajnc@chem.ox.ac.uk (A.K.); tharindi.panduwawala@chem.ox.ac.uk (T.D.P.); 2 Latvian Institute of Organic Synthesis, LV-1006 Riga, Latvia; anete.parkova@gmail.com (A.P.); peteris@osi.lv (P.T.); 3 School of Chemistry, University of Leeds, Leeds LS2 9JT, UK; ricky.cain@evotec.com (R.C.); C.W.G.Fishwick@leeds.ac.uk (C.W.G.F.); 4 Jacobs University Bremen gGmbH, 28759 Bremen, Germany; julesjoseph.philippe@gmail.com (J.P.); malcolm.page@antibiotic-research.ch (M.G.P.P.)

**Keywords:** antibiotic resistance, β-lactam antibacterial, bicyclic boronate inhibitors, VNRX-5133/taniborbactam, vaborbactam, metallo- and serine-β-lactamase inhibition, transition state analogue

## Abstract

Resistance to β-lactam antibacterials, importantly via production of β-lactamases, threatens their widespread use. Bicyclic boronates show promise as clinically useful, dual-action inhibitors of both serine- (SBL) and metallo- (MBL) β-lactamases. In combination with cefepime, the bicyclic boronate taniborbactam is in phase 3 clinical trials for treatment of complicated urinary tract infections. We report kinetic and crystallographic studies on the inhibition of AmpC, the class C β-lactamase from *Escherichia coli*, by bicyclic boronates, including taniborbactam, with different C-3 side chains. The combined studies reveal that an acylamino side chain is not essential for potent AmpC inhibition by active site binding bicyclic boronates. The tricyclic form of taniborbactam was observed bound to the surface of crystalline AmpC, but not at the active site, where the bicyclic form was observed. Structural comparisons reveal insights into why active site binding of a tricyclic form has been observed with the NDM-1 MBL, but not with other studied β-lactamases. Together with reported studies on the structural basis of inhibition of class A, B and D β-lactamases, our data support the proposal that bicyclic boronates are broad-spectrum β-lactamase inhibitors that work by mimicking a high energy ‘tetrahedral’ intermediate. These results suggest further SAR guided development could improve the breadth of clinically useful β-lactamase inhibition.

## 1. Introduction

**Figure 1:**
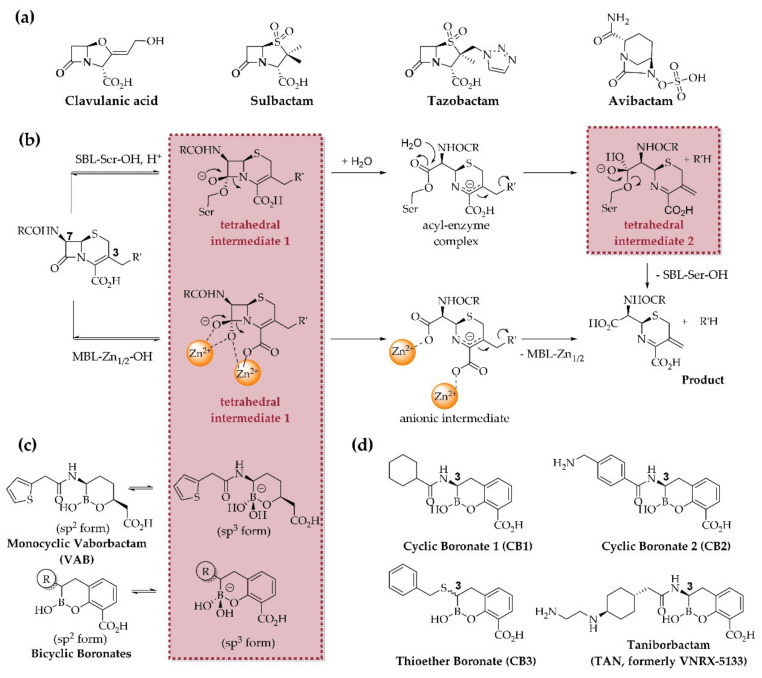
Mechanistic basis for β-lactamase inhibition by (bi)cyclic boronates. (**a**) clinically used β-lactamase inhibitors; (**b**) outline general mechanisms of SBLs and MBLs, exemplified by hydrolysis of a cephalosporin with elimination at C-3’. The tetrahedral intermediates, common to both SBL and MBL catalysis, may be mimicked by the sp^3^ form of (bi)cyclic boronates; (**c**) equilibria between sp^2^- and sp^3^-hybridized forms of mono- and bi-cyclic boronates; (**d**) structures of bicyclic boronate β-lactamase inhibitors cyclic boronate 1 (CB1), cyclic boronate 2 (CB2), the thioether boronate (CB3), and taniborbactam (TAN, formerly VNRX-5133). Note that the CB3 used in this study contained a mixture of the (3*S*)- and (3*R*)-enantiomers (~1:3, respectively) [16].

The β-lactams are amongst the most important antibiotics [1], but their widespread use is challenged by resistance, substantially through the global dissemination of β-lactamases [2,3]. There are two structural/mechanistic groups of β-lactamases—the nucleophilic serine-β-lactamases (SBLs: Ambler classes A, C, D) and the zinc dependent metallo-β-lactamases (MBLs: Ambler class B) (Figure 1b) [4]. Treatment of bacterial infections exhibiting resistance via some SBLs, particularly class A enzymes, was substantially advanced by the co-administration of a β-lactam antibiotic with a β-lactam-based SBL inhibitor, i.e., clavulanic acid [5,6], sulbactam [7], or tazobactam [8] (Figure 1a). However, these inhibitors are inactive, or are insufficiently active, against most class C SBLs, class A carbapenemases, and class B MBLs [3]. The clinical introduction of avibactam and avibactam derivatives such as relebactam [9] was an important step in more broadly combating SBLs because avibactam is active against class A β-lactamases (including carbapenemases), class C β-lactamases, and some class D β-lactamases [10]. Avibactam is also important because it demonstrates the clinical viability of non-β-lactam-based β-lactamase inhibition [11]. However, there is emerging SBL-mediated resistance to avibactam [12,13] and MBLs can hydrolyze avibactam, albeit slowly [14], suggesting that its future use may be compromised by the evolution of new β-lactamases [14,15].

By contrast with the successes in inhibiting SBLs, to date, there are no clinically useful MBL inhibitors [3]. There is thus interest in development of non-acylating β-lactamase inhibitors, especially those exhibiting dual-action SBL and MBL inhibition. Boron-based compounds have long been known as SBL and, more recently, MBL inhibitors [17]. After early work on acyclic boronic acids, recent efforts have focused on cyclic boronates. One such compound, the (predominantly) monocyclic boronic acid vaborbactam [18] (VAB, Figure 1c) which is approved for treatment of complicated urinary tract infections (cUTI) in co-administration with meropenem [19]. Vaborbactam is a relatively potent inhibitor of class A ESBLs and the KPC carbapenemases, but is not, at least usefully, active against tested MBLs and clinically relevant class C and D SBLs [19]. By contrast, boronates that are predominantly bicyclic in solution, are able to inhibit many, but not all, of the tested representatives of all four Ambler β-lactamase classes [20,21]. The bicyclic boronates are proposed to work by binding in a manner analogous to that of the tetrahedral intermediate(s) likely involved in β-lactam catalyzed hydrolysis by both SBLs and MBLs [20,21] (Figure 1b). Taniborbactam (TAN, formerly VNRX-5133, Figure 1d) has passed phase 1 clinical testing [22] and is currently in phase 3 clinical testing in combination with cefepime (registration No. NCT03840148 at ClinicalTrials.gov). TAN has been shown to successfully inhibit SBLs and MBLs *in vitro* [23,24,25,26], and to restore cefepime’s activity *in vivo* against a class A SBL (CTX-M-14) producing strain of *Klebsiella pneumoniae* in a neutropenic lung model of infection as well as CTX-M-15 expressing *E. coli* in ascending urinary tract infections in mice [26].

Many β-lactamases are encoded by plasmids, but the AmpC type β-lactamases that are present in most *Escherichia coli* strains are chromosomally encoded [27]. While normally expressed at low levels, mutations in the *ampC* gene promoter and/or attenuator regions can lead to constitutive hyperproduction of AmpC in *E. coli* [28,29]. This is clinically relevant because *E. coli* is a common Gram-negative pathogen, which is often responsible for bloodstream infections [30].

Structural data on the modes of action of bicyclic boronate β-lactamase inhibitors are relatively limited [20,21,24,26,31,32,33]. Work to date indicates that more than one side chain binding mode can occur, making structure-guided optimization challenging. Work on the B1 subfamily MBL NDM-1 has shown partial cyclisation of the TAN acylamino side chain to form a tricyclic structure, as an observed bound at the NDM-1 active site by protein crystallography [24]. The extent to which such tricyclisation contributes to β-lactamase inhibition more generally is unclear.

To enable future efforts on the optimization of bicyclic boronates, we report crystallographic and kinetic studies on inhibition of the clinically important class C AmpC β-lactamase from *Escherichia coli* (AmpC*_EC_*) by TAN, a previously reported model bicyclic boronate 2 (CB2) [20,21,34], and a thioether bicyclic boronate derivative (CB3) that we have recently shown to have enhanced potency against some SBLs and MBLs *in vitro* [16,35]. Together with previously reported studies [20,21,24,26,31,32,33], the crystallographic results support the proposal that bicyclic boronates mimic the tetrahedral intermediate(s) common to both SBL and MBL catalysis. The results indicate scope for the modification of the C-3 side chain of bicyclic boronates to improve potency towards AmpC type β-lactamases and inform on the likelihood of tricycle formation.

## 2. Materials and Methods

### 2.1. Materials

CB2, TAN, and CB3 were prepared as previously described [16,20,24]; VAB was purchased from Cayman Chemical (An Arbor, MI, USA). FC-5 was prepared as previously described [36].

### 2.2. Enzyme Production

Recombinant AmpC from *Escherichia coli* was produced according to a modification of the reported protocol [37]. In brief, AmpC*_EC_* was produced using the pAD7 vector [38] in *E. coli* W3110 cells using 2TY media supplemented with 12.5 mg/mL tetracycline. Cells were grown overnight at 37 °C, harvested via centrifugation (10 min, 12,000× *g*, 4 °C), resuspended in 50 mL lysis buffer (20 mM Tris pH 7.5, 10 mM MgCl_2_, 50 µg/mL DNAse I), then lyzed via sonification. Subsequent chromatography steps employed ÄKTA (GE Healthcare, Chicago, IL, USA) FPLC machines. The resultant supernatant was dialyzed with three buffer exchanges of 300 mL 10 mM Tris pH 6.75, then loaded onto a SP Sepharose (GE Healthcare, Chicago, IL, USA) cation exchange column and gradually eluted using a gradient of 10–75 mM Tris pH 7.0. Fractions containing the purified protein were pooled and concentrated to 20 mg/mL using an Amicon Ultra (MilliporeSigma, Burlington, MA, USA) centrifugal filter (15 mL, 10kDa MWCO). The protein was highly purified (>95%) as judged by SDS-PAGE and electrospray ionization mass spectrometry analyses (observed mass: 39,551 Da, calculated mass: 39,551 Da).

### 2.3. Kinetics

All kinetic measurements were carried out in competition assays using the fluorescent substrate FC-5 [36] under steady-state conditions monitoring hydrolysis, in 100 mM phosphate buffer pH 7.5 supplemented with 0.01% (*v/v*) Triton X-100 (Assay Buffer). Fluorescence was measured using a PHERAstarFS (BMG Labtech, Aylesbury, United Kingdom) plate-reader, recording emission spectra at λ_ex_ = 380 nm and λ_em_ = 460 nm. IC_50_s were determined after 10 min incubation time of 500 pM AmpC*_EC_* with varying concentrations of inhibitors and assayed using 5 µM FC-5. Nonlinear regression analyses were carried out using GraphPad Prism V. 5.04 (GraphPad Software, San Diego, CA, USA).

As described for avibactam inhibition [11], the kinetics of bicyclic boronate inhibition of β-lactamases can be described assuming a two-step, reversible inhibition model:

(1) $$E+I\;\begin{array}{c} \overset{\;k_{1}\;}{\to }\\ \underset{k-1}{\leftarrow } \end{array}\;E:I\;\begin{array}{c} \overset{\;k_{2}\;}{\to }\\ \underset{k-2}{\leftarrow } \end{array}\;E-I$$

with *E:* enzyme; *I*: inhibitor; *k*_1_: association rate constant; *k*_−1_: dissociation rate constant; *k*_2_: binding rate constant, and *k*_−2_: recyclization rate constant.

To obtain the pseudo first-order rate constant *k*_obs_ and the apparent inhibition constant *K*_iapp,_ the rate of FC-5 (5 µM) hydrolysis by AmpC*_EC_* (100 nM) was measured in the presence of varying concentrations of the inhibitor. Time-courses were then first fitted to Equation (2) to give the observed initial rate constants *k*_obs_ as previously described [39]:

(2) $$P=V_{s}t+\left(V_{0}-V_{S}\right)\frac{\left(1-e^{-k_{obs}t}\right)}{k_{obs}}+P_{0}$$

with P: formed product, proportional to fluorescence signal; $P_{0}$: initial fluorescence; $V_{S}$: velocity of no-inhibitor control; and $V_{0}$: velocity of no-enzyme control to estimate a fully inhibited enzyme.

Plotting the resultant *k*_obs_ values against the inhibitor concentration [I] (Figure S1b) and fitting to Equation (3) gives the apparent second-order rate constant (*k*_2_/*K’*)

(3) kobs=k2+k2K′∗ I

with *K*’ representing the equilibrium coefficient of AmpC*_EC_* inhibition in the presence of FC-5 in the set concentration of 5 µM.

This can then be corrected using the Michaelis constant (*K_M_*) of FC-5 for AmpC*_EC_* and the FC-5 concentration [S] according to Equation (4) to give the second-order rate constant (*k*_2_/*K*):

(4) k2K=k2K′∗ SKM+1

The same data were used to determine the apparent inhibition constant *K*_iapp_ [40]. The reciprocals of the initial rates were plotted against inhibitor concentration (Figure S1a), giving a straight line for which the value of the intercept divided by the slope gives *K*_iapp_’. From this, *K*_iapp_ can be obtained after correction with the *K_M_* for FC-5 according to Equation (5):

(5) $$K_{iapp}=\frac{K_{iapp}^{’}}{1+\left(\frac{\left[S\right]}{K_{M}}\right)}$$

Off rates (*k*_-2_ or *k*_off_) were measured using the jump-dilution method [41] (Figure S1c). AmpC*_EC_* (1 µM) was incubated with the respective inhibitor (10 µM for TAN, CB2 and CB3, and 100 µM for VAB) for 30 min at room temperature, then diluted 100,000 fold in the Assay Buffer (final enzyme concentration: 10 pM) and immediately assayed with 25 µM FC-5. The data were fitted to Equation (2) with $V_{0}$ in this case representing the velocity of the no-enzyme control and $V_{S}$ representing the initial velocity of uninhibited enzyme.

The half-life of the enzyme-inhibitor complex t_1/2_ is given by Equation (6):

(6) $$t_{1/2}=\frac{\ln\left(2\right)}{k_{off}}$$

### 2.4. Antimicrobial Susceptibility Testing

Minimum inhibitory concentrations (MICs) were determined by broth microdilution in triplicate and interpreted using published Clinical and Laboratory Standards Institute (CLSI) guidelines [22].

Ceftazidime (CAZ) was tested alone (0.25–256 μg mL^−1^) against DH5α *Escherichia coli* and DH5α *E. coli* containing the pAD7-AmpC*_EC_* plasmid and in combination with CB2, CB3, TAN, and VAB (all tested at a fixed concentration of 4 μg mL^−1^) using the DH5α *E. coli* strain containing the pAD7-AmpC*_EC_* plasmid [38].

### 2.5. Crystallization Experiments, X-Ray Data Collection and Processing

Crystallization plates (low reservoir Intelli-Plate 93-3, Art Robbins Instruments, Sunnyvale, CA, USA) were set up with a Phoenix RE Drop setter instrument (Art Robbins Instrument, Sunnyvale, CA, USA). Crystals were grown via the vapor diffusion technique at room temperature. To obtain AmpC*_EC_*-TAN and AmpC*_EC_*-CB3 complex crystals, crystals of apo-AmpC*_EC_* were grown in Condition A, comprising 200 nL AmpC_EC_ (20 mg/mL in 50 mM Tris pH 7.5), mixed with 200 nL Precipitant Solution (10 mM zinc chloride, 100 mM MES pH 6.0 and 20% (*w/v*) PEG 6000). Crystals grew at room temperature over 2–3 days, were transferred into a well solution supplemented with approximately 20 mM inhibitor and incubated for 15 and 10 min, respectively. These AmpC_EC_ crystals are highly sensitive to DMSO and prolonged soaking times (>15 min). Soaking was successful for TAN and CB3, but, in the case of CB2, only low occupancy was observed for CB2 following soaking under the same conditions. A single crystal of the AmpC*_EC_*-CB2 complex was obtained via co-crystallization using Condition B, comprising 200 nL AmpC*_EC_* (18 mg/mL in 50 mM Tris pH 7.5) and CB2 (20 mM), mixed with 200 nL Precipitant Solution (150 mM HEPES, 60% (*v/v*) 2-methyl-2,4-pentanediol (MPD)). Crystals grew at room temperature over 1–2 weeks. Due to the less robust crystallization system and the lower resolution of the data obtained (see Table S1), the co-crystallization approach was not explored for TAN and CB3. Crystals were cryo-cooled and then stored in liquid nitrogen. Datasets from single crystals were collected using the i03 and i24 MX beamlines at the Diamond Light Source (Table S1). Structures were solved by molecular replacement in Phaser [42] using PDB ID 1IEM [43] as the starting model. Alternating cycles of refinement using PHENIX [44] and model building using Coot [45] were performed until R_work_ and R_free_ converged. Coordinates and structure factors have been deposited in the Protein Data Bank. PDB IDs are 6T3D, 6YEO, 6YPD and 6YEN for the crystal structure of AmpC from *Escherichia coli* in its apo-form and in complex with CB2, CB3, and TAN, respectively.

## 3. Results and Discussion

### 3.1. Kinetic Studies of AmpC_EC_ Inhibition by Bicyclic Boronates

To investigate the importance of different side chains at the C-3 position (equivalent to C-6 of penicillins or C-7 of cephalosporins) of the bicyclic boronate core for inhibition of AmpC*_EC_*, steady-state kinetic assays (Table 1) were carried out using the fluorescent reporter substrate FC-5 [36]. All three tested bicyclic boronates showed enhanced potency for AmpC*_EC_* inhibition compared to the monocyclic boronate VAB (*K*_iapp_ ~ 1–4 µM for the bicyclic boronates, compared to 19 µM for VAB). Interestingly, the thioether CB3 was slightly more potent against isolated AmpC*_EC_* than TAN and the structurally similar compound CB2 (*K*_iapp_ 1 µM for CB3, compared to 3 µM and 4 µM for CB2 and TAN, respectively). The increased potency of CB3 likely results from accelerated binding (*k*_2_/*K* ~ 224 × 10^3^ M^−1^ s^−1^ compared to ~ 81/86 × 10^3^ M^−1^ s^−1^ for CB2/TAN and ~ 7 × 10^3^ M^−1^ s^−1^ for VAB). However, dissociation of CB3 is equally accelerated (21 × 10^−3^ s^−1^, compared to 3 × 10^−3^ s^−1^ for CB2/TAN and 0.7 × 10^−3^ s^−1^ for VAB), resulting in a shorter half-life of the enzyme-inhibitor complex for CB3. This proposal is supported by pIC_50_ measurements showing equivalent potency of all three tested bicyclic boronates after a 10 min pre-incubation period with AmpC*_EC_* (pIC_50_s of 7.5 for CB2, TAN, and CB3 compared to 6.3 for VAB).

### 3.2. Microbiology Experiments Confirm Potential of (Bi)cyclic Boronates to Inhibit AmpC_EC_ in Cells

We then investigated the activity of VAB, TAN, CB2 and CB3 (fixed at 4 µg mL^−1^) in combination with ceftazidime (CAZ) against *E. coli* DH5α producing AmpC*_EC_* (Table 2). Production of AmpC*_EC_* substantially increased the extent of CAZ resistance (MIC 256 µg mL^−1^ compared to 1 µg mL^−1^ without the AmpC*_EC_* expressing plasmid); all the tested inhibitors significantly restored CAZ activity against the AmpC*_EC_* expressing strain. Despite the high potency of CB3 observed in the kinetic analyses against isolated AmpC*_EC_*, it was less potent than CB2, TAN or VAB in restoring CAZ activity in the engineered strain, i.e., CB3 reduced the MIC only to 4 µg mL^−1^, whereas the other inhibitors restored the activity of CAZ to ≤ 1 µg mL^−1^.

### 3.3. AmpC_EC_ Crystal Structures with Bicyclic Boronates Give New Insights into Structural Basis of Inhibition

To investigate the structural basis of AmpC*_EC_*-inhibition by the three bicyclic boronates (CB2, TAN, and CB3), we obtained crystal structures of their complexes with recombinant AmpC*_EC_*. The structures of AmpC*_EC_* in complex with TAN (1.42 Å resolution, PDB ID: 6YEN) and CB3 (1.60 Å resolution, PDB ID: 6YPD) were obtained by soaking of apo-AmpC*_EC_* crystals. A structure of the complex of AmpC*_EC_* with CB2 (2.03 Å resolution, PDB ID: 6YEO) was obtained by co-crystallization (see Materials and Methods). Apo-AmpC*_EC_* crystallized with a single chain in the asymmetric unit (space group *P*4_3_32), while the asymmetric unit of the AmpC*_EC_*-CB2 co-crystal complex contains four molecules of the β-lactamase (space group *P*222_1_).

**Figure 2:**
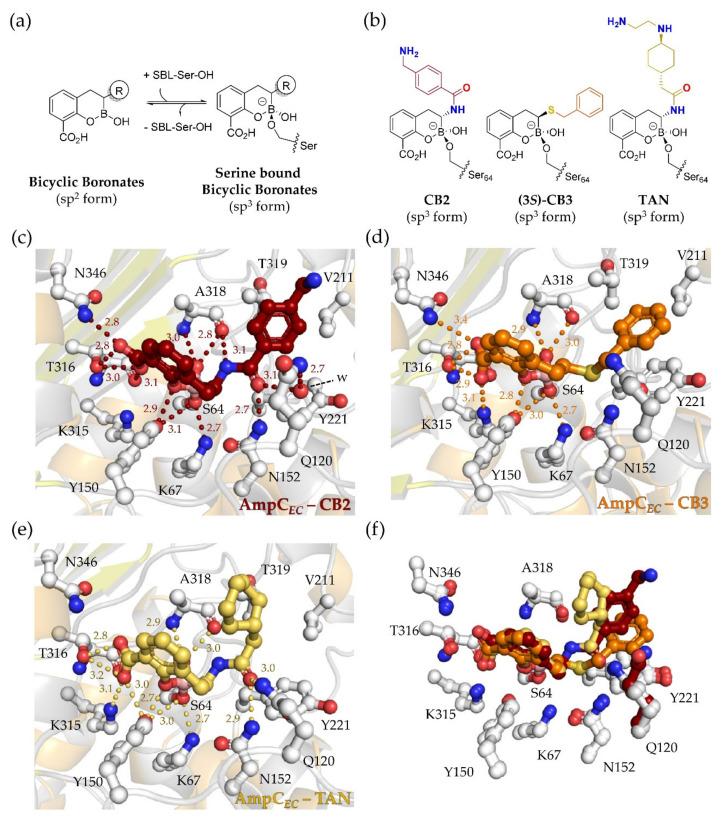
Structural basis for AmpC*_EC_* inhibition by bicyclic boronates. (**a**) reaction between sp^2^-hybridized bicyclic boronates and SBLs give a serine-bonded anionic sp^3^-hybridized species; (**b**) structures of serine-bonded sp^3^ forms of the bicyclic boronates; (**c**–**e**) active site views of complexes of AmpC*_EC_* with CB2, CB3, and TAN. Hydrogen-bonding interactions are shown in colored dashes, distances are in Å; (**f**) overlay of active site views, color coding as in (**c**–**e**). Note that the Q120 side chain is rotated in AmpC*_EC_*-CB2 structure (highlighted in dark red); it is unclear whether this is induced by binding of CB2, or due to the crystallization conditions (see text).

The overall fold of the AmpC*_EC_*-boronate complexes is very similar to that of the apo-AmpC*_EC_* structure (PDB ID: 6T3D; main chain RMSDs with respect to the apo-structure: 0.39, 0.15, 0.07 Å for CB2, TAN, and CB3, respectively, for chain A). In all three structures, analysis of the electron density maps for the AmpC*_EC_* active site indicates that the bicyclic boronates CB2, TAN, and CB3 have reacted with the nucleophilic S64 to form an anionic tetrahedral species in which the boron is sp^3^ hybridized (Figure 2), as observed for other bicyclic boronate SBL complex structures [20,21,26,31,32,33]. The bicyclic core of all three inhibitors in complex with AmpC*_EC_* is bound in a similar manner to that observed for cyclic boronate 1 (CB1, Figure 1d) in complex with the *Pseudomonas aeruginosa* AmpC (PDB ID: 6I30 [32]). In none of these cases did any of the active site bound bicyclic boronates form a tricyclic structure, as was observed in crystallographic studies of the MBL NDM-1-TAN complex [24].

The aryl carboxylic acid of the bicyclic boronates is positioned similarly to that of the monocyclic inhibitor VAB as observed in complex with the *Enterobacter cloacae* AmpC (PDB ID: 4XUX [18]) and that of CB1 observed in complex with *P. aeruginosa* AmpC [32]. The aryl carboxylic acid of all the bicyclic boronates is involved in hydrogen-bonding or electrostatic interactions with active site residues K315, T316, and N346. The boron-linked hydroxyl group engages in hydrogen-bonding interactions with the backbone NH and oxygen of A318, as observed for the equivalent S345/338 residues in the *P. aeruginos*a AmpC-CB1 and *E. cloacae* AmpC-VAB complex structures [18,32].

As observed in the *P. aeruginos*a AmpC-avibactam [46] and *P. aeruginos*a AmpC-relebactam [9,47] complex structures, the amide nitrogen and carbonyl group of the TAN acylamino side chain are positioned to make hydrogen-bonding interactions with active site residues, i.e., Q120, N152, and A318. The cyclohexyl ring of TAN is directed out of the active-site R1 pocket (Figure 2e); only weak density was observed for the likely disordered terminal alkyl-amine side chain, which was excluded from the model.

AmpC*_EC_* was co-crystallized with CB2 giving crystals with four molecules in each asymmetric unit, all of which manifested clear and continuous electron density for the covalently bound CB2 at the active site (Figure S2). The overall folds and orientations of active site residues and CB2 (Figure S2d) are very similar in all four chains (main chain RMSD of Chain B, C and D in relation to chain A: 0.31, 0.28, and 0.33 Å, respectively). Binding of the bicyclic boronate core of CB2, together with that of the amido side chain group portion, is very similar to that observed for TAN (CB3 has a different type of side chain).

Interestingly, in the CB2 structure, the side chain of Q120 is directed away from the active site in all four molecules in the asymmetric unit (Figure 2c); Q120 interacts with a water molecule (Figure 2c, ‘w’, observed in all chains of the ASU), which in turn interacts with the carbonyl oxygen of the acylamino CB2 side chain. In the apo structure of AmpC*_EC_* in condition A (PDB ID: 6T3D), the Q120 side chain is oriented towards the active site; however, the apparently weak density and elevated B-factors for the Q120 side chain indicate partial disorder. The reorientation of the Q120 side chain relative to the apo-enzyme observed in the AmpC*_EC_*-CB2 complex is not manifest in the structure of the AmpC*_EC_*-TAN complex. Although the AmpC*_EC_*-CB2 complex was co-crystallized using a different crystallization condition than for the two other structures reported here (see Materials and Methods), because the Q120 side chain does not interact with residues of other AmpC*_EC_* molecules in the crystalline lattice, reorientation due to formation of crystal contacts seems unlikely. In a crystal structure of AmpC*_EC_*, obtained by soaking with CB2, partial density for the bound CB2 was observed in the active site, though reorientation of the Q120 side chain was not observed (data not shown, because of poor electron density for the CB2 C-3 side chain). It is thus unclear whether the different orientation of Q120 compared to apo-AmpC*_EC_* is due to the different crystallization conditions or due to a specific feature of CB2 binding. The positioning of the Q120 side chain could impact on the potency of inhibition by bicyclic boronates containing an amido side chain, as in CB2 and TAN.

A structure of the AmpC*_EC_*-CB3 complex was obtained by soaking of apo crystals of the β-lactamase. CB3 does not contain the amido C-3 side chain, but instead features a benzyl-thioether side chain (Figure 1d) [16]. CB3 was synthesized as a mixture of the (3*S*)- and (3*R*)-isomers with the (3*R*)-isomer in excess (~24:76 (*S*:*R*) ratio) [16]. Although binding of low levels of the (3*R*)-isomer cannot be excluded, only evidence for binding of the (3*S*)-CB3 was observed at the active site of AmpC*_EC_*. The thioether side chain is partially disordered, as manifested by ~ 50% elevated B-factors compared to the CB3 average. The thioether is positioned to make hydrophobic π-stacking interaction with Y221 (~ 4 Å distance between Y221 and CB3 benzyl rings) and hydrophobic interactions with the side chains of V211 and T319 in the R1 pocket (Figure 2d). These hydrophobic residues located on the AmpC*_EC_* Ω-loop (residuess 185-225) are also present in the *P. aeruginosa* AmpC, rationalizing the high potency of CB3 against both AmpC*_EC_* and *P. aeruginosa* AmpC and likely other class C SBLs, as reported here and elsewhere [16]. Notably, mutations in the Ω-loop, e.g., deletion of Y221, have been observed to widen the substrate specificity and also confer ceftazidime-avibactam resistance [12]. Such AmpC variants may be less susceptible to bicyclic boronates like CB3 and are thus a potential source of resistance to such inhibitors. This is of particular concern given that mutations in the Ω-loop have been linked to reduced susceptibility of cefepime against AmpC-expressing *E. coli* [48], which is the β-lactam proposed for use in combination with TAN and which is not efficiently turned-over by the wildtype AmpC*_EC_*.

**Figure 3:**
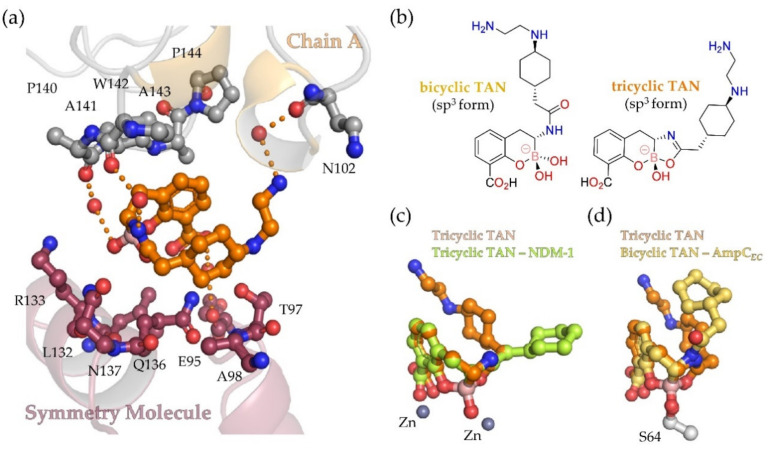
Comparison of bi- and tri-cyclic forms of sp^3^ hybridized taniborbactam in complex with AmpC*_EC_*. (**a**) The tricyclic form of TAN as observed at the interface between symmetry related AmpC*_EC_* molecules along a three-fold rotation axis (see Figure S3a for overview, PDB ID 6YEN). Residues within 6 Å of TAN are shown as grey/purple sticks, hydrogen-bonding interactions as orange dashes, and waters as red spheres; (**b**) structures of proposed bi- and tricyclic sp^3^ forms of TAN; (**c**) overlay of the ‘interface’ tricyclic TAN (AmpC*_EC_*, orange) with active site bound tricyclic TAN (NDM-1, green); (**d**) overlay of the ‘interface’ tricyclic TAN (AmpC*_EC_*, orange) with active site bound bicyclic TAN bonded to AmpC*_EC_* S64 (yellow). Note, with the MBL NDM-1 (PDB ID: 6RMF), both the bicyclic and the tricyclic forms were observed in one chain of the crystallographic dimer (chain A, shown here), while, in the second chain, only the tricyclic form was observed [24].

Interestingly, apart from covalent modification of S64, analysis of the electron density map in the structure obtained by soaking AmpC*_EC_* with TAN clearly reveals a second TAN molecule at the interface between chain A and symmetry related molecules (Figure S3a). The second TAN molecule adopts a U-shaped conformation in which its side chain folds over the bicyclic core (Figure 3); it is positioned to form hydrogen-bonding interactions with multiple water molecules that interact with the backbone carbonyls of P140, A141, and N102, and to make hydrophobic interactions with the side chains of A143 and A98 in a symmetry related molecule (Figure 3a).

Notably, with the surface bound TAN molecule, its C-3 amido side chain is rotated relative to the conformation observed for bicylic TAN at the active site, positioning the carbonyl derived oxygen atom in close proximity to the boron. Based on the short distance between the refined carbonyl oxygen and the boron (1.5 Å), this molecule of TAN was thus modelled in a tricyclic form, as shown in Figure 3b. The 5-membered ring of this TAN molecule was modelled in its unsaturated form based on its planar conformation and the angle between the carbonyl-derived oxygen, carbon- and amide-derived nitrogen atoms (122°). The observation of tricyclic TAN on the surface of AmpC*_EC_* indicates the viability of its formation in solution; reaction of acyl-amino side chains to form a ring onto a proximate boronic acid has synthetic precedent [49]. An analogous tricycle has also been observed in the complex formed between the B1 MBL NDM-1 and TAN [24]. Overlay of the tricyclic boronate core observed with AmpC*_EC_* and that observed in the active site of NDM-1 shows a strong resemblance (Figure 3c); however, the side chains adopt different conformations in the two structures.

**Figure 4:**
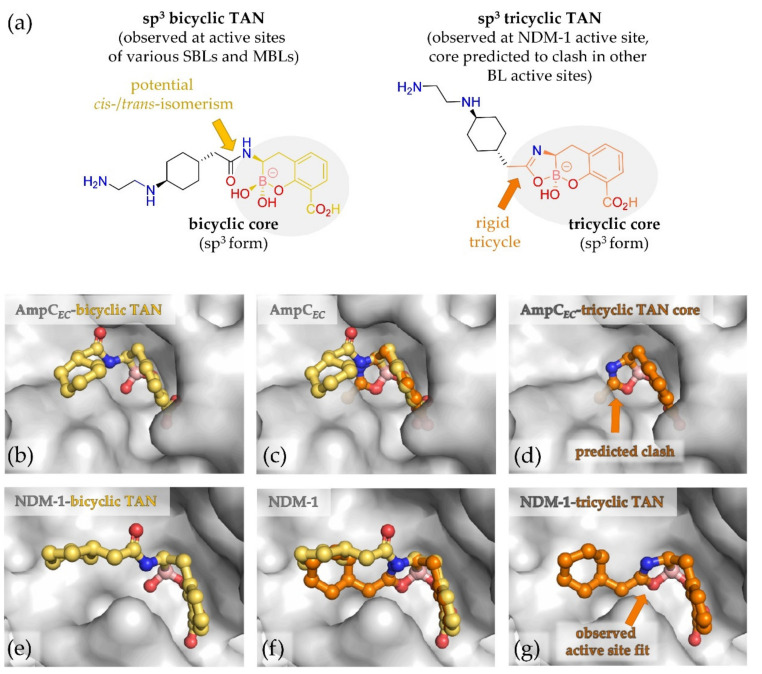
Tricyclic TAN likely cannot bind at the AmpC*_EC_* active site due to a steric clash of the putative tricyclic core and side chain. (**a**) Structural comparison of tricyclic and bicyclic forms of TAN; (**b**–**g**) overlay of tricyclic TAN core (orange, not crystallographically observed at AmpC*_EC_* active site) with bicyclic TAN (yellow) crystallographically observed bound to active site S64 of AmpC*_EC_* (PDB ID: 6YEN) reveals a likely steric clash of the rigid tricycle in the AmpC*_EC_* active site as well as active sites of other β-lactamases (Figure S5). By contrast, both tricyclic (orange) and bicyclic (yellow) forms of TAN have been crystallographically observed at the active site of the B1 MBL NDM-1 (PDB ID: 6RMF) [24]. Note, in both structures, that the terminal amine of the side chain was disordered and therefore excluded from the model; (**b**) observed conformation of bicyclic TAN at the AmpC*_EC_* active site; (**c**) alignment of tricyclic TAN core to bicyclic TAN observed at the AmpC*_EC_* active site; (**d**) putative steric clash of tricyclic TAN in the AmpC*_EC_* active site based on the overlay in c). Note the apparently flexible parts of the side chain are not shown, but would make a clear steric clash with the active site; (**e**) observed conformation of bicyclic TAN at the NDM-1 active site; (**f**) overlay of tricyclic TAN and bicyclic TAN, both as observed at the NDM-1 active site; (**g**) observed conformation of tricyclic TAN at the NDM-1 active site.

Importantly, overlays of the bicyclic portions of the tricyclic TAN core with the bicyclic TAN reveals a steric clash for a potential tricycle, including its side chain, at the AmpC*_EC_* active site (Figure 4). This analysis is consistent with the observed bicyclic structure at the AmpC*_EC_* active site and indicates that it is unlikely that the boronates studied can bind to the AmpC*_EC_* active site in the tricyclic form (at least without conformational change). Further comparisons suggest that the other SBLs, for which crystallographic studies are reported in complex with the bicyclic form of TAN (i.e., KPC-2 [31] (PDB ID 6TD1), CTX-M-15 [26] (PDB ID 6SP6), OXA-10 [24] (PDB ID 6RTN)), will also not accommodate the tricyclic core, assuming a conserved general binding mode of the bicycle and no conformational changes (Figure S5).

Although there are substantial differences in the active site chemistry of the SBLs and MBLs, crystallographic analysis on the class B1 MBL VIM-2 [26] (PDB ID: 6SP7) shows that the bicyclic conformation of TAN as observed in SBLs is maintained in binding to this clinically relevant MBL (Figure S5). In the case of NDM-1, active site binding of both bicyclic and tricyclic TAN was observed as shown in Figure 4e–g (~75% of the active sites were occupied by the tricyclic and ~25% by the bicyclic form) [24]. By contrast, overlay of the tricyclic form of TAN with the bicyclic form of TAN as observed in the active site of VIM-2 reveals a steric clash (Figure S5). This implies that the tricyclic form of TAN is likely relevant to the inhibition of some (NDM-1), but not all MBLs, assuming a conserved binding mode for the tri-/bi-cycle core and a lack of substantial conformational changes. It should be noted that recent work has shown other tricyclic boronates, i.e., those containing a cyclopropyl group fused to the C2/C3 carbons of the bicyclic boronate potently inhibit both SBLs and MBLs [50], though the cyclopropyl ring methylene projects in a different orientation to the ‘third’ ring of the tricyclic ring system that can be formed with TAN (Figure S6).

## 4. Conclusions

Following the clinical application of boronic acids as proteasome inhibitors for treatment of myeloma, boronic acids/boronates have become more common in medicinal chemistry [17]. In the antibacterial field, the monocyclic boronate vaborbactam has been approved for clinical use as an inhibitor of class A and some class C SBLs [19]. More recent work has demonstrated that the scope of boronates as β-lactamase inhibitors can be extended to multiple SBLs and MBLs by integration into a bicyclic scaffold [20,21], as present in TAN [26] (Figure 1d), which is currently in late stage clinical trials. Our kinetic and structural studies with TAN and other bicyclic boronate derivatives (CB2, CB3) with different C-3 side chains reveal their potential for inhibition of the class C AmpC serine-β-lactamase from *Escherichia coli*.

Our results further support the potential of bicyclic boronates as unusually broad-spectrum inhibitors of all SBL types and, at least B1 type, MBLs. It is possible that the potential of cyclic boronates as clinically useful inhibitors of two or more mechanistically distinct groups of enzymes will extend to other families, such as proteases. Improvements in methods for their preparation should enable more systematic SAR. In this regard, it may be that the incorporation of the boron into a ring system that limits conformations and modes of reactivity may both enhance potency and reduce unwanted modes of reaction (e.g., oligomerization) compared to acyclic boronic acids.

Combined with previous reports, our three crystal structures of bicyclic boronates with AmpC*_EC_* reveal remarkable similarity between the binding modes of the 6,6-core ring system of the bicyclic boronates to SBLs from class A (e.g., CTX-M-15 [21,26], L2 [33], and KPC-2 [31]), class D (e.g., OXA-10 [20,24]) and the class C AmpC from *Pseudomonas aeruginosa* [32] as well as the penicillin-binding protein-5 (PBP-5) from *Escherichia coli* [20]. The results thus support the proposal that mimicking high-energy intermediates common to different types of enzymes can enable their inhibition by the same inhibitor. Note that the reported bicyclic boronates tend to be weaker PBP inhibitors (and hence antibiotics) than SBL/B1 MBL inhibitors [20,21]; this may reflect the fact that, although they react with β-lactams, PBPs are not optimized to catalyze their hydrolysis. Alternatively, it may be that optimized functionalization of the core bicyclic boronate will enable potent PBP inhibition. The combined results show that, in the case of SBL/MBL inhibition by bicyclic boronates, there is scope for (C-3) side chain optimization, which may further broaden the scope of β-lactamase inhibition, including to multiple B2/B3 type MBLs. Optimization in potency will need to be carried out in parallel with that at a microbiological level, as has been done for TAN with respect to improved cell penetration [26]. Interestingly, although VAB was substantially less potent than TAN against isolated AmpC*_EC_*, it performed equally well against our AmpC bearing engineered *E. coli* strain as TAN (Table 2), possibly reflecting either improved penetration or reduced efflux.

Together with previous reports showing enhanced activity of CB3 against class D SBLs, as well as certain B2 and B3 MBLs compared to TAN and CB2 [16], our results suggest the thioether CB3 could be a promising start for further optimization towards a broad-spectrum, dual SBL and MBL inhibitor. Selective binding of the minor (3*S*)-isomer indicates that stereoselective synthesis might further benefit AmpC*_EC_* inhibition. Further optimization will need to address the relatively low potency of CB3 in cells, which could reflect potential poor cell penetration of CB3 due to its lipophilicity. CB3 has a predicted logP of 4.42; studies have shown that compounds with relatively low logPs (e.g., CB2, TAN and VAB with predicted logPs of 2.09, 1.50, and 1.86, respectively) manifest improved penetration of Gram-negative bacteria [51], leaving further scope for optimization of the thioether side chain, e.g., by addition of an amino group as in TAN [26].

The observation of an apparently weakly bound tricyclic form of TAN on the surface of AmpC*_EC_* is of interest from a boronate chemistry perspective as it potentially further exemplifies the ability of boron-based inhibitors to interchange between different forms in aqueous solution. It should be noted that, in both the cases where a tricyclic boronate form has been observed crystallographically, i.e., with AmpC*_EC_* and the MBL NDM-1, the crystallization conditions were acidic (pH 6.0 and 5.8 [24], respectively). Thus, the tricycle could be formed in aqueous solution during crystallization; such formation in aqueous solution is precedented in small molecule chemistry [49]. Comparison of SBL and MBL structures with tri- and bi-cyclic boronates suggests most SBL active sites will not readily accommodate tricylic structures of the type reported here and previously [24]. Although tricyclic TAN likely does not significantly contribute to inhibition of AmpC*_EC_* and related SBLs, at least one β-lactamase (NDM-1) can bind a tricyclic form at its active site (at least in crystals) [24], thus the ability of boronate type inhibitors to interchange between different forms has the potential to extend the range of β-lactamases usefully inhibited by them.

## Figures and Tables

**Table 1:** Kinetic analyses of AmpC*_EC_* inhibition by (bi)cyclic boronates *in vitro*. *K*_iapp_ values and pseudo first-order rates (*k*_2_/*K*) were determined by assaying 100 nM AmpC*_EC_* with 5 µM FC-5 [36]. *k*_off_ rates were determined after jump-dilution (100,000 fold) of AmpC*_EC_* (1 μM) that had been pre-incubated with cyclic boronates (10 µM for TAN, CB2 and CB3 and 100 µM for VAB) at room temperature for 30 min, then assayed using 25 μM FC-5. pIC_50_s were obtained from assays using 500 pM AmpC*_EC_* and 5 µM FC-5 following a 10 min inhibitor pre-incubation at room temperature. Buffer: 50 mM Tris, pH 7.5, 0.01% (*v/v*) Triton X-100. Data were analyzed as described in Materials and Methods.

Inhibitor	*K*_iapp_ (μM)	k2K (M^−1^ s^−1^) × 10^3^	*k*_off_ (s^−1^) × 10^−3^	t_1/2_ (min)	pIC_50_
**VAB**	19.1 ± 1.32	6.55 ± 0.46	0.69 ± 0.03	16.8 ± 0.7	6.32 ± 0.03
**TAN**	3.73 ± 0.50	85.6 ± 9.2	2.54 ± 0.51	4.55 ± 0.91	7.53 ± 0.02
**CB2**	3.26 ± 0.40	81.0 ± 6.4	2.77 ± 0.27	4.18 ± 0.41	7.49 ± 0.01
**CB3**	1.16 ± 0.14	224 ± 21	20.9 ± 12.8	0.55 ± 0.34	7.53 ± 0.02

**Table 2:** Microbiology analyses with vaborbactam (VAB), taniborbactam (TAN), cyclic boronate 2 (CB2) and cyclic boronate 3 (CB3). All inhibitors were tested at a fixed concentration of 4 µg mL^−1^.

		Minimum Inhibitory Concentration Ceftazidime (µg mL^−1^)				
Strain	Plasmid	CAZ	CAZ (+ **VAB)**	CAZ (+ **TAN**)	CAZ (+ **CB2**)	CAZ (+ **CB3**)
DH5α	-	1	-	-	-	-
DH5α	pAD7 AmpC*_EC_*	256	1	1	0.5	4

## Supplementary Materials

The following are available online at https://www.mdpi.com/2218-273X/10/6/899/s1, Table S1: Data collection and refinement statistics of AmpC*_EC_* crystals. Figure S1: Kinetic characterization of reversible AmpC*_EC_* inhibition by cyclic boronates. Figure S2: Views from a crystal Structure of AmpC*_EC_* in complex with CB2. Figure S3: Views from a crystal structure of AmpC*_EC_* in complex with TAN. Figure S4: Views from a crystal structure of AmpC*_EC_* in complex with CB3. Figure S5: The tricyclic form of TAN likely cannot bind to some β-lactamases including members from all Ambler classes. Figure S6: Structures of the tricyclic form of TAN and QPX7728.
