# Supplementary material for: Bicyclic Boronates as Potent Inhibitors of AmpC, the Class C β-Lactamase from Escherichia coli
Source: Biomolecules. 2020 Jun 12;10(6):899. doi: 10.3390/biom10060899 (PMC7356297; doi:10.3390/biom10060899)
Supplement: Supplementary file 1 [file biomolecules-10-00899-s001.pdf]

Supplementary Information

Bicyclic Boronates as Potent Inhibitors of AmpC, the Class C β-Lactamase from *Escherichia coli*

Pauline A. Lang [1], Anete Parkova [2,†], Thomas M. Leissing [1,†], Karina Calvopiña [1], Ricky Cain [3,‡], Alen Krajnc [1,‡], Tharindi D. Panduwawala [1], Jules Philippe [4], Colin W. G. Fishwick [3], Peteris Trapencieris [2], Malcolm G. P. Page [4], Christopher J. Schofield [1,*] and Jürgen Brem [1,*]

[1] Department of Chemistry, University of Oxford, Chemistry Research Laboratory, OX1 3TA Oxford, United Kingdom

[2] Latvian Institute of Organic Synthesis, LV-1006 Riga, Latvia; peteris@osi.lv (P.T.)

[3] School of Chemistry, University of Leeds, LS2 9JT Leeds, United Kingdom; C.W.G.Fishwick@leeds.ac.uk (C.W.G.F.)

[4] Jacobs University Bremen gGmbH, Life Sciences & Chemistry, 28759 Bremen, Germany; malcolm.page@antibiotic-research.ch (M.G.P.P.)

* Correspondence: christopher.schofield@chem.ox.ac.uk (C.J.S.); jurgen.brem@chem.ox.ac.uk (J.B.)

† These authors contributed equally to this work.

‡ These authors contributed equally to this work.

Table 1. Data collection and refinement statistics of $AmpC_{EC}$ crystals.

Datasets	$AmpC_{EC}$-Apo (PDB ID: 6T3D)	$AmpC_{EC}$-Taniborbactam (PDB ID: 6YEN)	$AmpC_{EC}$-CB3 (PDB ID: 6YPD)
**Data Collection**			
Beamline (Wavelength, Å)	DLS I03 (0.9763)	DLS I03 (0.9787)	DLS I03 (0.9762)
Detector	Eiger2 XE 16M	Pilatus 6M-F	Eiger2 XE 16M
Data Processing	Xia2 3dii	Xia2 autoPROC	dials
Space group	$P\ 4_3\ 3\ 2$	$P\ 4_3\ 3\ 2$	$P\ 4_3\ 3\ 2$
Cell dimensions			
a,b,c (Å)	139.2, 139.2, 139.2	137.3, 137.2, 137.2	139.0, 139.0, 139.0
α, β, γ (°)	90, 90, 90	90, 90, 90	90, 90, 90
No. of molecules/ASU	1	1	1
No. reflections	73865 (5399)*	83449 (4083)*	60941 (2948)*
Resolution (Å)	49.21-1.50 (1.54-1.50)*	97.04-1.42 (1.44-1.42)*	138.97-1.60 (1.63-1.60)*
R_{meas}(I)	0.104 (6.300)*	0.135 (7.265)*	0.166 (4.538)*
I/σI	29.3 (1.2)*	26.6 (1.0)*	22.1 (1.3)*
CC-half	1.00 (0.51)*	1.0 (0.4)*	1.0 (0.7)*
Completeness (%)	100 (100)*	100 (100)*	100 (99.9)*
Multiplicity	76.6 (77.9)*	76.9 (78.6)*	77.3 (67.4)*
Wilson B value (Å^2)	23.97	21.32	23.35
**Refinement**	PHENIX	PHENIX	PHENIX
R_{work}/R_{free}	0.1582/0.1777	0.1442/0.1687	0.1709/0.1874
No. atoms			
- Enzyme	2871	2941	2919
- Bound Inhibitor	-	24	22
- Unbound Inhibitor	-	28	-
- Water	343	349	376
Average B-factors	34.5	28.4	31.0
- Enzyme	33.2	26.7	29.4
- Bound Inhibitor	-	33.6	57.0
- Unbound Inhibitor	-	51.2	-
- Water	44.7	39.4	40.2
R.m.s deviations			
- Bond lengths (Å)	0.007	0.008	0.009
- Bond angles (°)	0.820	1.132	0.979

*Highest resolution shell in parentheses.

Table 1. continued.

Datasets	**AmpC$_{EC}$-CB2 (PDB ID: 6YEO)**
**Data Collection**	
Beamline (Wavelength, Å)	DLS I24 (0.9686)
Detector	Pilatus3 6M
Data Processing	Xia2 dials
Space group	$P\ 2\ 2\ 2_1$
Cell dimensions	
a,b,c (Å)	100.7, 179.6, 99.7
α, β, γ (°)	90, 90, 90
No. of molecules/ASU	4
No. reflections	117532 (5616)*
Resolution (Å)	100.68-2.03 (2.06-2.03)*
R_{meas}(I)	0.368 (0.999)*
I/σI	8.7 (2.7)*
CC-half	1.0 (0.6)*
Completeness (%)	100 (96.7)*
Multiplicity	13.0 (13.1)*
Wilson B value (Å^2)	24.11
**Refinement**	PHENIX
R_{work}/R_{free}	0.1715/0.2027
No. atoms	
- Enzyme	10950
- Bound Inhibitor	101
- Unbound Inhibitor	-
- Water	864
Average B-factors	29.1
- Enzyme	28.3
- Bound Inhibitor	34.7
- Unbound Inhibitor	-
- Water	36.0
R.m.s deviations	
- Bond lengths (Å)	0.002
- Bond angles (°)	0.612

*Highest resolution shell in parentheses.

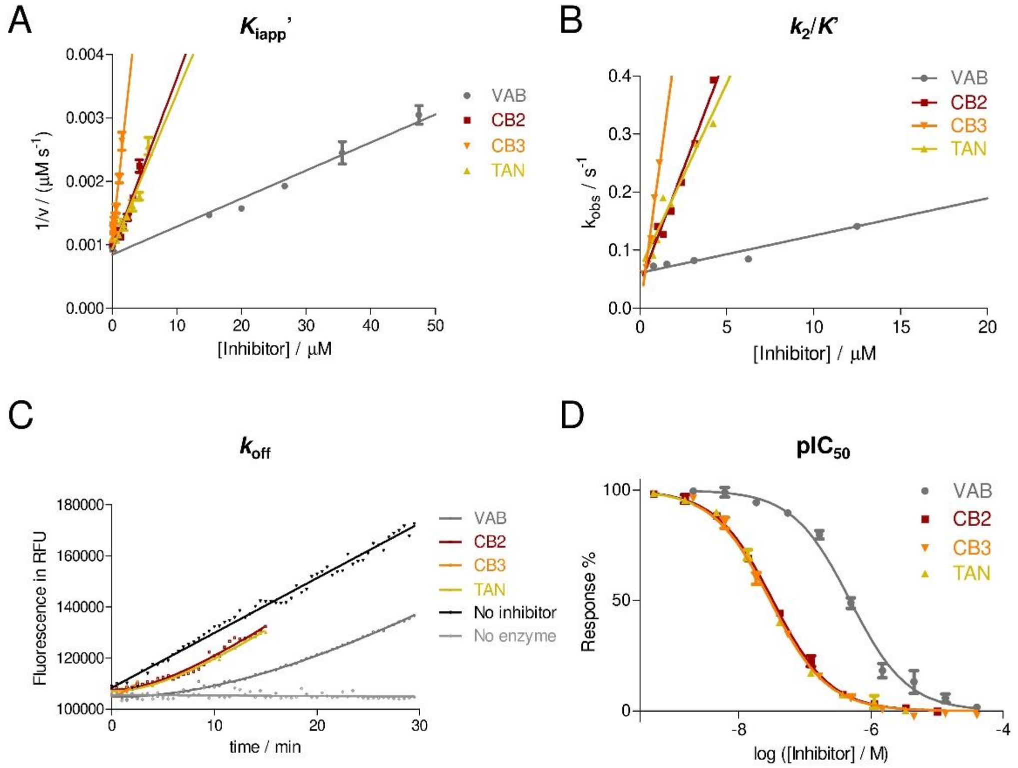

Figure 1. Kinetic characterisation of reversible AmpC_{EC} inhibition by cyclic boronates. (**A**) Determination of K_{iapp} value of AmpC_{EC} inhibition by cyclic boronates. (**B**) Pseudo first-order rate (k_2/K) determination for AmpC_{EC} (100 nM) with TAN, CB2, or CB3. (**C**) Dissociation after 'jump-dilution' of an AmpC_{EC} solution (10 μM) pre-incubated with cyclic boronates (10 μM for TAN, CB2 and CB3 and 100 μM for VAB) at room temperature for 30 min, then diluted 100.000 fold and assayed with FC-5[1] (25 μM). (**D**) Dose-response curves for AmpC_{EC} (500 pM) after 10 min inhibitor pre-incubation at room temperature, assayed using FC-5[1] (5 μM). Buffer: 50 mM Tris, pH 7.5, 0.01 % (*v*/*v*) Triton-X 100.

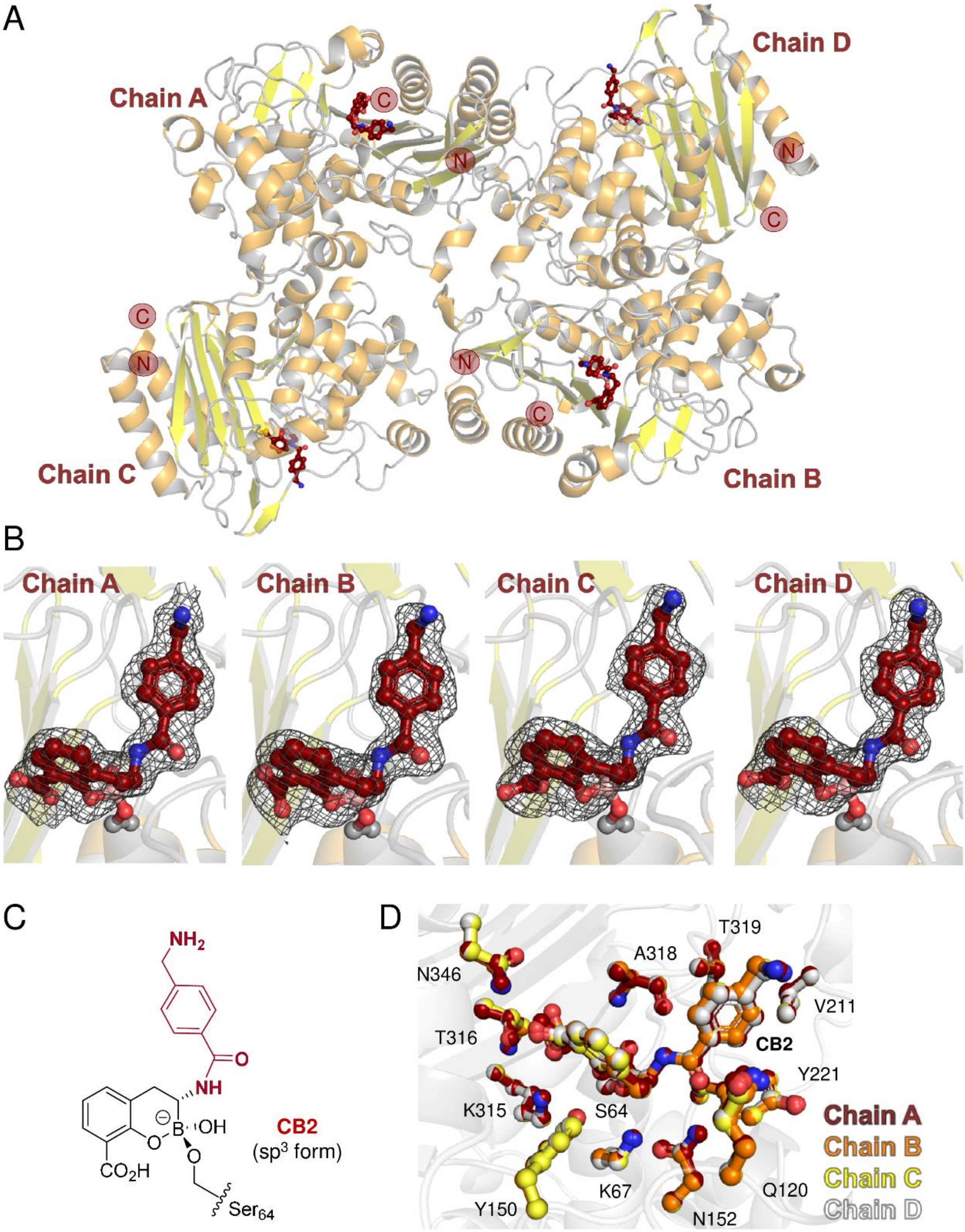

Figure 2. Views from a crystal structure of AmpC*EC* in complex with CB2. (**A**) Overview of the asymmetric unit (ASU) containing chains A-D. (**B**) mFo-DFc polder OMIT maps[2] contoured at 3.0 σ around CB2. (**C**) Structure of bound CB2 in its sp^3 form. (**D**) Overlay of color coded active site residues and ligands in chains A–D s in the ASU shows no differences in the orientations of these residues and CB2.

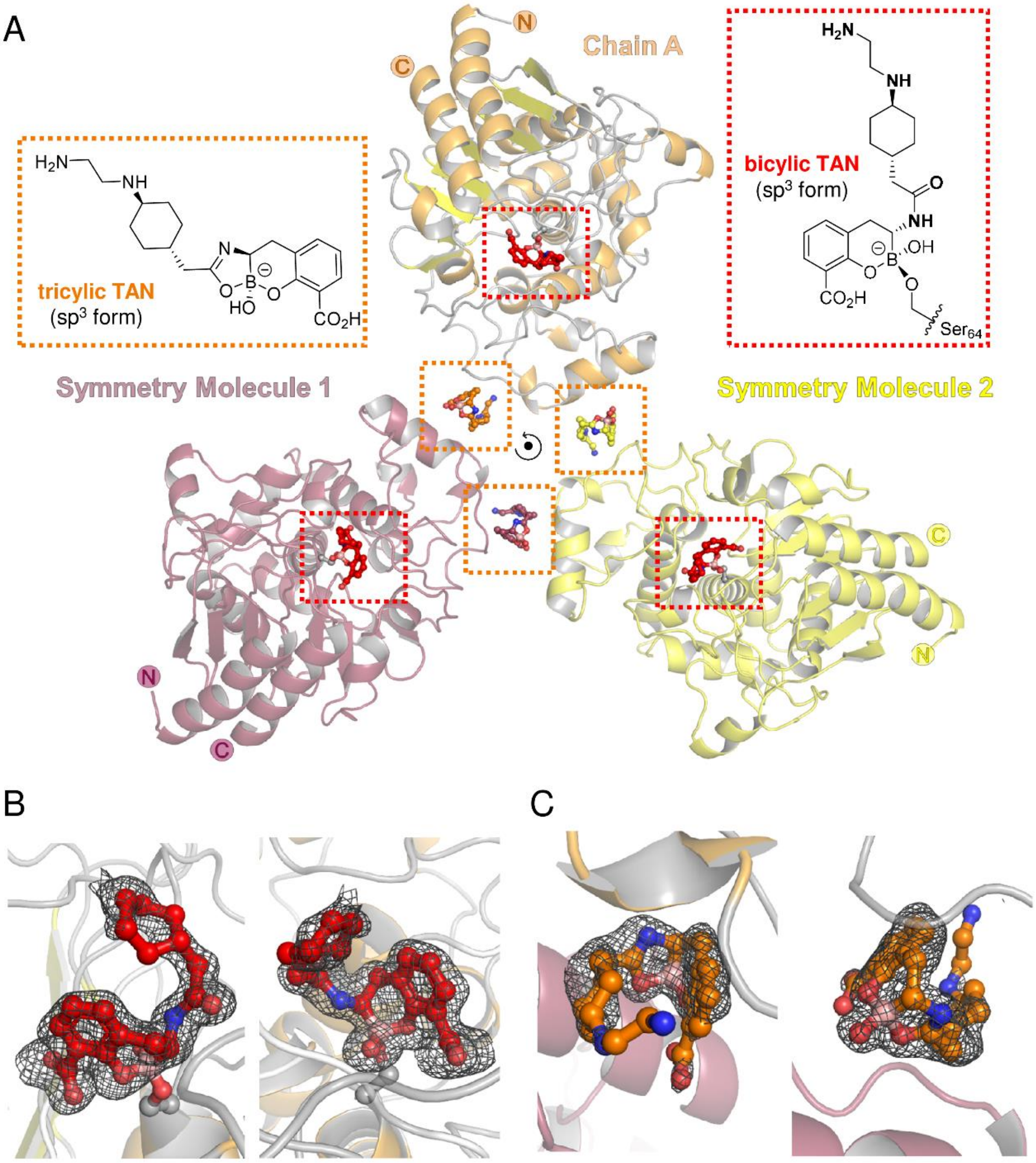

Figure 3. Views from a crystal structure of AmpCEC in complex with TAN. (**A**) Overview of symmetry related molecules around the 3-fold rotation axis, showing bicyclic TAN (red) bonded to the nucleophilic Ser64 and the position of tricyclic TAN (color coded by AmpCEC molecule) at the monomer interfaces. (**B**) Views of mFo-DFc polder OMIT maps[2] contoured at 3.0 σ and carved around bicyclic TAN bonded to Ser64. (**C**) Views of mFo-DFc polder OMIT maps[2] contoured at 2.5 σ around tricyclic TAN, which binds at the interface between AmpC molecules.

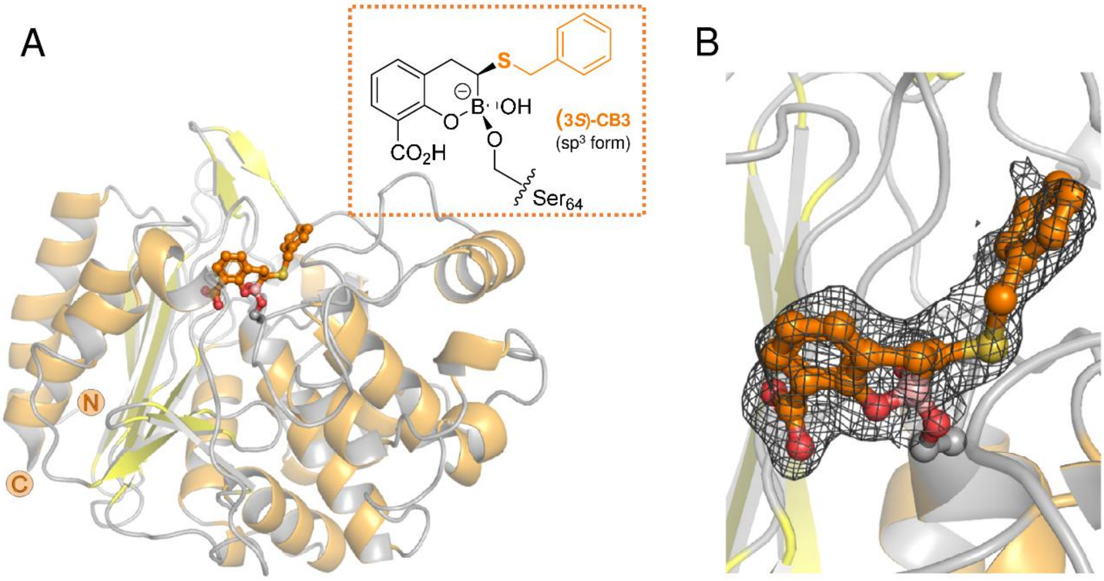

Figure 4. Views from a crystal structure of AmpC*EC* in complex with CB3. (**A**) Overview of AmpCEC fold, showing CB3 (orange) bound to active site. (**B**) mFo-DFc polder OMIT maps[2] contoured at 3.0 σ and carved around CB3 bonded to Ser64.

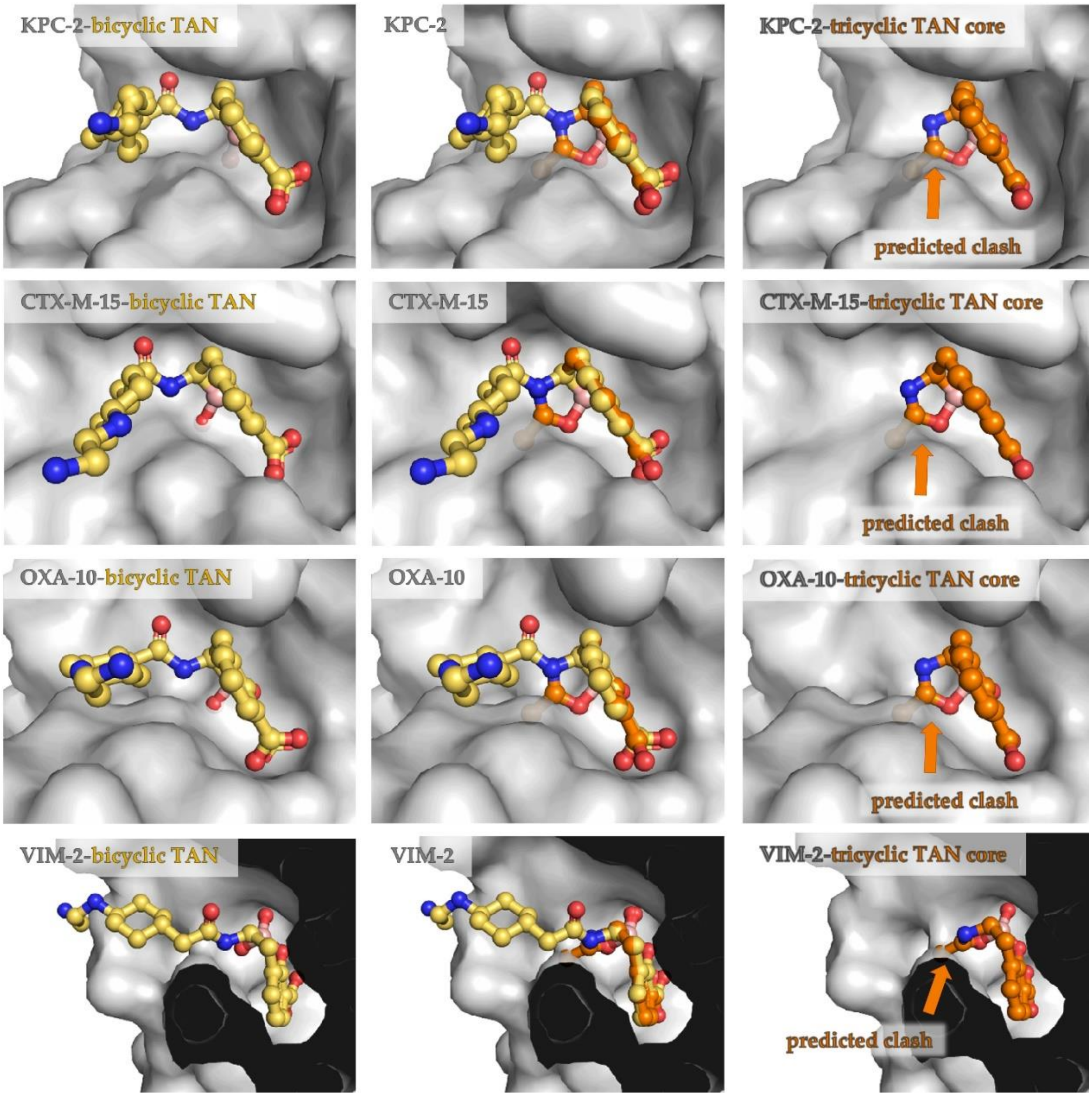

Figure 5. The tricyclic form of TAN likely cannot bind to some β-lactamases including members from all Ambler Classes. Overlays of the tricyclic TAN core (orange, as observed at AmpC$_{EC}$ surface (Figure 4, PDB ID: 6YEN)) with bicyclic TAN complexes (yellow) as crystallographically observed at the active sites of KPC-2[3] (PDB ID 6TD1), CTX-M-15[4] (PDB ID 6SP6), OXA-10[5] (PDB ID 6RTN), and VIM-2[4] (PDB ID 6SP7) reveals a likely steric clash of the rigid tricycle in the shown active sites. Left column: Observed conformations of the bicyclic form of TAN at the β-lactamase active sites; Middle column: Overlays of the tricyclic TAN and bicyclic TAN forms at the indicated active sites; Right column: The putative steric clashes of tricyclic TAN in the indicated β-lactamase active sites based on the overlays in the middle column. Note, the flexible parts of the tricyclic TAN inhibitor sidechain are not shown, but would make a clear steric clash with the active site.

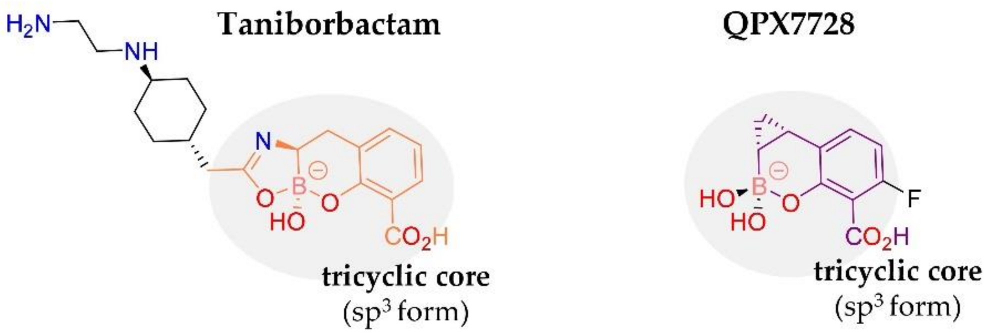

Figure 6. Structures of the tricyclic form of TAN and QPX7728. Shown are the tricyclic form of TAN as observed at AmpC*EC* surface and QPX7728[6] which has a cyclopropyl ring fused to its bicyclic core.

References

1. van Berkel, S. S.; Brem, J.; Rydzik, A. M.; Salimraj, R.; Cain, R.; Verma, A.; Owens, R. J.; Fishwick, C. W. G.; Spencer, J.; Schofield, C. J., Assay platform for clinically relevant metallo-β-lactamases. *J Med Chem* **2013,** *56*, 6945–6953.
2. Liebschner, D.; Afonine, P. V.; Moriarty, N. W.; Poon, B. K.; Sobolev, O. V.; Terwilliger, T. C.; Adams, P. D., Polder maps: improving OMIT maps by excluding bulk solvent. *Acta Crystallogr D Struct Biol* **2017,** *73*, 148–157.
3. Tooke, C. L.; Hinchliffe, P.; Krajnc, A.; Mulholland, A. J.; Brem, J.; Schofield, C. J.; Spencer, J., Cyclic boronates as versatile scaffolds for KPC-2 β-lactamase inhibition. *RCS Med Chem* **2020**, 1–7.
4. Liu, B.; Trout, R. E. L.; Chu, G.-H.; McGarry, D.; Jackson, R. W.; Hamrick, J. C.; Daigle, D. M.; Cusick, S. M.; Pozzi, C.; De Luca, F.; et al., Discovery of Taniborbactam (VNRX-5133): A Broad-Spectrum Serine- and Metallo-β-lactamase Inhibitor for Carbapenem-Resistant Bacterial Infections. *J Med Chem* **2020,** *63*, 2789–2801.
5. Krajnc, A.; Brem, J.; Hinchliffe, P.; Calvopina, K.; Panduwawala, T. D.; Lang, P. A.; Kamps, J.; Tyrrell, J. M.; Widlake, E.; Saward, B. G.; et al., Bicyclic Boronate VNRX-5133 Inhibits Metallo- and Serine-β-Lactamases. *J Med Chem* **2019,** *62*, 8544–8556.
6. Hecker, S. J.; Reddy, K. R.; Lomovskaya, O.; Griffith, D. C.; Rubio-Aparicio, D.; Nelson, K.; Tsivkovski, R.; Sun, D.; Sabet, M.; Tarazi, Z.; et al., Discovery of Cyclic Boronic Acid QPX7728, an Ultrabroad-Spectrum Inhibitor of Serine and Metallo-β-lactamases. *J Med Chem* **2020**, 1–17.
